# Non-negligible impacts of urbanization on spatiotemporal variations of infectious disease: a case study of hemorrhagic fever with renal syndrome epidemics in China

**DOI:** 10.1186/s40249-025-01366-w

**Published:** 2025-10-16

**Authors:** Qi Wei, Hongyan Ren, Liang Lu, Runhe Shi

**Affiliations:** 1 Key Laboratory of Geographic Information Science, Ministry of Education, East China Normal University, Shanghai, 200241 China; 2 State Key Laboratory of Resources and Environmental Information System, Institute of Geographic Sciences and Natural Resources Research, Chinese Academy of Sciences, Beijing, 100101 China; 3 School of Geographic Sciences, East China Normal University, Shanghai, 200241 China; 4 Joint Laboratory of Environmental Remote Sensing and Data Assimilation, East China Normal University, Shanghai, 200241 China; 5 National Key Laboratory of Intelligent Tracking and Forecasting for Infectious Diseases, National Institute for Communicable Disease Control and Prevention, Chinese Center for Disease Control and Prevention, Beijing, 102206 China

**Keywords:** Hemorrhagic fever with renal syndrome, Meteorological factor, Multidimensional urbanization, Joinpoint regression, Geodector analysis, China

## Abstract

**Background:**

Hemorrhagic fever with renal syndrome (HFRS) poses a significant public health concern in China. However, the spatiotemporal patterns and underlying drivers of its transmission are not fully understood. This study aims to investigate spatiotemporal heterogeneity of HFRS incidence at the city level and explore its potential influencing factors.

**Methods:**

Joinpoint regression was utilized to analyze city-level HFRS incidence data (*n* = 314 cities, 2005–2021) collected from the National Infectious Disease Surveillance System. Furthermore, we employed the Geodetector method to identify the potential driving factors from a set of meteorological, vegetation, and urbanization variables.

**Results:**

The results from Joinpoint regression analysis revealed an overall declining trend in city-level HFRS incidence across China from 2005 to 2021. Of the cities analyzed, 126 showed an upward trend [the average annual percent change,(AAPC) > 0], 176 a downward trend (AAPC < 0), and 12 remained stable (AAPC = 0). Notably, upward-trend cities were predominantly concentrated in South China. Geodetector analysis indicated that selected climatic and vegetation factors accounted for 19–56% of the spatiotemporal heterogeneity in HFRS incidence, whereas urbanization factors explained only 3–5%. However, synergistic interactions between temperature and urbanization-related variables (i.e., land-use, economic, and demographic dimensions) significantly enhanced their explanatory power, particularly in upward-trend cities, where the combinations increased explanatory capacity by 124–184%.

**Conclusion:**

In summary, while climatic and vegetation factors remain the primary drivers of the spatiotemporal heterogeneity of HFRS epidemics in China, urbanization also exerts non-negligible influence on city-level incidence. This research offers valuable insights for public health authorities to strengthen their intervention capabilities against this disease.

**Graphical Abstract:**

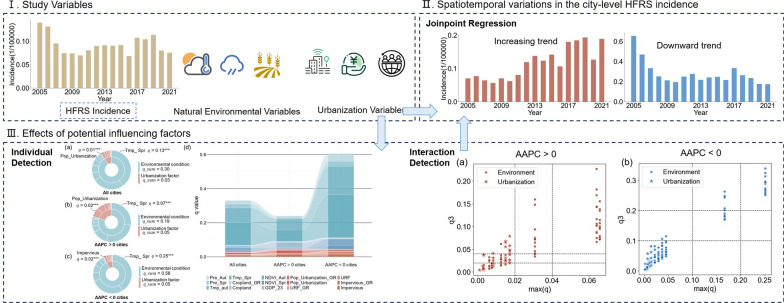

## Background

Hemorrhagic fever with renal syndrome (HFRS), a rodent-borne zoonotic disease caused by *Hantaviruses* [1], has been reported in over 70 countries. China bears the highest global burden, accounting for 70%–90% of cases worldwide [2, 3]. Recent declines in HFRS incidence are largely attributed to comprehensive preventive measures, including public health education and publicity, rodent surveillance and control, and vaccination programs [4, 5]. Nevertheless, this disease remains a serious public health threat, particularly in emerging endemic regions in China [2, 6].

HFRS transmission is highly dependent on natural environmental factors. Temperature and precipitation critically influence both virus survival and rodent dynamics, with moderate conditions amplifying transmission risks, while extreme meteorological events suppress host activity and viral persistence [3, 5]. Vegetation coverage (e.g., the maize and peanut cultivation areas) enhances habitat suitability and food availability for rodent populations, thereby elevating zoonotic exposure risks [6–8]. Furthermore, HFRS exhibits distinct seasonality patterns driven by pathogen-host ecology: Seoul virus outbreaks peak in spring (March–June) primarily in *Rattus norvegicus*-dominant regions, whereas Hantaan virus epidemics surge in autum/winter (October–January) in areas dominated by *Apodemus agrarus* [9, 10]. Consequently, HFRS prevalence in China demonstrates marked spatial heterogeneity, with hyperendemic foci predominant clustered in the southwestern, northeastern, eastern, and central regions, whereas arid northwest zones (e.g., Qinghai, Xinjiang) report fewer cases [4, 9].

As a multidimensional process encompassing economic transition, land-use transformation, and population redistribution, China’s urbanization presented marked spatiotemporal disparity, including phase-specific developmental stages coexisted with regionally divergent patterns driven by imbalanced resource allocation and institutional reforms [11]. Land-use intensification (e.g., construction-driven habitat encroachment) elevates rodent-human interface risks, while economic advancement mitigates such interactions by reducing environmental contamination and resource availability for rodents [4, 12, 13]. Most existing studies commonly focusing on typical HFRS outbreak zones at small scales or provincial levels, by which the impacts of urbanization have been assessed in northeastern China (e.g., Heilongjiang Province [1, 14]) and northwestern China (e.g., Shaanxi Province [6, 15]). Owing to stage-dependent heterogeneity and developmental disparities in China’s urbanization, these findings exhibit significant variations across major regional categories.

Previous studies on the spatiotemporal variations in HFRS epidemics and their influencing factors have strongly supported nationwide surveillance and prevention and control and improved our understanding of the prevalence of this disease in China [3, 16]. However, multidimensional characteristics of China’s urbanization, particularly their complex impacts on the spatiotemporal heterogeneity of HFRS epidemics, remain inadequately elucidated.

Thus, we conducted this study to (1) characterize the spatiotemporal Heterogeneity of HFRS across 314 cities from 2005 to 2021 through long-term trend analysis, (2) identify the relative contributions of climatic, vegetative, and urbanization drivers to epidemic patterns, and (3) assess synergistic amplification effects from urbanization-environment interactions.

## Methods

### Data collection

The city-level HFRS incidence data were sourced from the National Infectious Disease Surveillance System from 2005 to 2021. Based on previous studies and data availability, we gathered 22 potential independent variables (Table 1), containing two aspects: environmental conditions and urbanization. The natural environmental variables comprised meteorological (temperature and precipitation), and vegetation such as cropland and normalized difference vegetation index (NDVI) factors. In China, the process of urbanization is often investigated by various indices, including the percentage of impervious area and urban-rural fringe zones indicating the land urbanization, the secondary and tertiary industry share of gross domestic production (GDP) for the economic urbanization, and the ratio of urban residents for the population urbanization [17–19]. These urbanization elements pose different impacts on the ecological surroundings of the rodents’ activities, the healthcare conditions of the local population, and the population proportions, respectively [20–23]. All independent variables were integrated into annual datasets for the period 2005–2021.

**Table 1:** Data sources and collection methodologies encompassed in this study

Data group	Selected variables	Data unit	Time scale	Resolution	Source
HFRS incidence	Incidence rate	1/100,000	Annual	City-level	The National Infectious Disease Surveillance System
Natural factors [24–27]	Tmp_Spr	Degree Celsius	Monthly	30 m	National Tibetan Plateau/Third Pole Environment Data Center (https://data.tpdc.ac.cn/)
	Tmp_Aut	Degree Celsius	Monthly	30 m	
	Prt_Spr	mm	Monthly	30 m	
	Prt_Aut	mm	Monthly	30 m	
	Cropland	%	Annual	30 m	The 30 m annual land cover dataset (https://zenodo.org/records/15853565)
	Cropland_GR	\	Annual	30 m	
	NDVI_Spr	\	Monthly	1 km	MOD13A3(https://ladsweb.modaps.eosdis.nasa.gov/missions-and-measurements/products/MOD13A)
	NDVI_Aut	\	Monthly	1 km	
Urbanization Factors [14, 28–32]	GDP_23	%	Annual	City-level	China city statistical yearbook
	Pop_Urbanization	%	Annual	City-level	
	Pop_Urbanization_GR	\	Annual	City-level	
	Impervious	%	Annual	30 m	The 30 m annual land cover dataset (https://zenodo.org/records/15853565)
	URF	%	Annual	30 m	
	Impervious_GR	\	Annual	30 m	Joinpoint methods based on Impervious
	URF_GR	\	Annual	30 m	

HFRS incidence: incidence of hemorrhagic fever with renal syndrome; GDP: gross domestic production; MOD13A3: monthly level 3 gridded vegetation indices product derived from moderate-resolution imaging spectroradiometer; Prt_Aut: mean autumn precipitation; Prt_Spr: mean spring precipitation; Tmp_Aut: mean autumn temperature; Tmp_Spr: mean spring temperature; GDP_23: GDP share of secondary and tertiary sectors; Pop_Urbanization_GR: growth rate of resident population urbanization rate; Pop_Urbanization: urbanization rate of the resident population; URF_GR: growth rate of urban–rural fringe area; URF: percentage of urban–rural fringe area; Impervious_GR: growth rate of impervious area; Impervious: percentage of impervious area; Cropland_GR: growth rate of cropland area; Cropland: percentage of cropland area; NDVI: normalized difference vegetation index; NDVI_Aut: mean autumn NDVI; NDVI_Spr: mean spring NDVI

Total of 314 cities with over 10 consecutive years of data from the HFRS dataset were selected for further examination in order to maintain the integrity of the analysis. Missing values were imputed using the average of data from the preceding and subsequent years.

Furthermore, our study calculated the growth rates (GR) of cropland area, impervious area, Percentage of urban-rural fringe area(URF) area, and urbanization rate, applying the following formula:

1 $$\begin{array}{c}y=\left({x}_{t+1}-{x}_{t}\right)/{x}_{t}\times 100\%\end{array}$$

where $y$ is GR values and ${x}_{t}$ denotes the value of an indicator (i.e., cropland area, imperious area, URF area, and urbanization rate) in year $t$.

### Temporal analysis of the city-level HFRS incidence

Joinpoint regression is commonly employed to explore the trends in the prevalence of disease so as to conduct a detailed assessment of its global and localized changes. In this study, a segmented log-linear model in Joinpoint Regression Program (version 5.1.0, https://surveillance.cancer.gov/joinpoint/) was performed on the annual incidence data of 314 cities from 2005 to 2021, by which the variations in the city-level HFRS incidence were spatiotemporally explored. The annual percent change (APC), which assessed the trend within each segment of the function, and the average annual percent change (AAPC), which represented the overall average trend in morbidity, were calculated as follows [33, 34]:

2 $$\begin{array}{c}APC=\left[\frac{{y}_{x+1}-{y}_{x}}{{y}_{x}}\right]\times 100=\left({e}^{{\beta }_{i}}-1\right)\times 100\end{array}$$

3 $$\begin{array}{c}AAPC=\left({e}^{\sum {\omega }_{i}{\beta }_{i}/\sum {\omega }_{i}}-1\right)\times 100\end{array}$$

where $y$ is the incidence rate, $x$ signifies the year of incidence, ${\omega }_{i}$ refers to the width of each interval after segmentation, and ${\beta }_{i}$ corresponds to the regression coefficient for each interval. Positive, negative, and zero AAPC values respectively indicate upward, downward, and stable trends at the global level. Additionally, we considered a range of 0 to 3 Joinpoints.

### Analysis of the relationship between HFRS incidence and selected factors

Given China’s significant regional heterogeneity in natural and socioeconomic conditions, we first conducted pearson correlation analysis at the city scale to quantify linear associations between selected factors and HFRS incidence, providing a basis for subsequent impact investigations.

The Geodetector method is widely applied to quantify spatial heterogeneity of dependent variables and to identify key drivers through factor detection (*q*-statistic), while its interaction detector module evaluates synergistic or antagonistic effects between independent variable pairs (http://geodetector.cn/). Geodetector was employed to quantify factor contributions (*q*-values) to HFRS incidence. Analyses covered: (1) long-term drivers (17-year pooled data) and (2) annual dynamics across all cities and AAPC-stratified subgroups (upward- and downward-trends). Stable-trend cities (AAPC = 0) were excluded as their invariant rates violate its variance-based detection principle.

The coupling between a single factor and HFRS incidence was determined using the following formula [35]:

4 $$\begin{array}{c}q=1-{\sum }_{h=1}^{L}{N}_{h}{\sigma }_{h}^{2}/\left(N{\sigma }^{2}\right)\end{array}$$

where $N$ and ${\sigma }^{2}$ are the number of sample units and variance of incidence per year, $h$ = 1, 2, …, and $L$ denotes the stratification of the variable, with at least 2 sample units in each stratification. $q$ acts as the measure of the explanatory power of the detection factor on the response factor (HFRS incidence rate), indicating a percentage ($q \times 100\%$), which in the range of $[\text{0,1}]$, where a higher value denotes stronger explanatory power. Specifically, a $q$ value of 0 signifies no coupling between the factor and incidence, while a $q$ value of 1 denotes that the morbidity is entirely determined by the factor.

The interaction detector identifies the ability of combined pairs in explaining the spatial heterogeneity of HFRS incidence under five scenarios [36]:

$$Enhance, nonlinear-:q\left({X}_{1} \cap { X}_{2} = {X}_{3}\right) > q\left({X}_{1}\right) + q\left({X}_{2}\right)$$

$$Independent:q\left({X}_{1} \cap {X}_{2} ={ X}_{3}\right) = q\left({X}_{1}\right) + q\left({X}_{2}\right)$$

$$Enhance, bi-:q\left({X}_{1} \cap {X}_{2} ={ X}_{3}\right) > Max(q\left({X}_{1}\right), q\left({X}_{2}\right))$$

$$Weaken, uni-:Min\left(q\left({X}_{1}\right), q\left({X}_{2}\right)\right) < q\left({X}_{1}\cap {X}_{2} ={ X}_{3}\right) < Max(q\left({X}_{1}\right),q\left({X}_{2}\right))$$

$$Weaken, nonlinear:q\left({X}_{1} \cap {X}_{2} = {X}_{3}\right) < Min(q\left({X}_{1}\right),q\left({X}_{2}\right))$$

The enhancement effect following the interaction of variables is evaluated using the $sq$ indicator [37]:

5 $$\begin{array}{c}sq = \frac{q\left({X}_{3}\right) - Max\left(q\left({X}_{1}\right),q\left({X}_{2}\right)\right)}{Max\left(q\left({X}_{1}\right),q\left({X}_{2}\right)\right)} * 100\%\end{array}$$

where $q({X}_{1})$ and $q({X}_{2})$ denotes the explanatory power of factors ${X}_{1}$ and ${X}_{2}$ on HFRS, $q({X}_{3})$ represents the influence of interaction detection of ${X}_{1}$ and ${X}_{2}$, $Min\left(q\left({X}_{1}\right),q\left({X}_{2}\right)\right)$ is the minimum of $q({X}_{1})$ and $q({X}_{2})$, and $Max(q\left({X}_{1}\right),q\left({X}_{2}\right))$ was the maximum of $q({X}_{1})$ and $q({X}_{2})$. $sq$ means the growth of the explanatory power of two variables before and after their interaction, $sq$= 0 indicates that the two variables are independent, and $sq$ > 0 reflects an enhancement effect after the interaction. A higher $sq$ value signifies a more pronounced enhancement effect.

Additionally, we ranked the level of explanatory power before ($q\left({X}_{1}\right),$ and $q\left({X}_{2}\right)$) and after ($q3$) the interaction, while $q3$ exceeding $Max\left(q\left({X}_{1}\right), q\left({X}_{2}\right)\right)$ was considered as a transitional combination.

## Results

### Spatiotemporal variations in the city-level HFRS incidence

**Fig.1:**
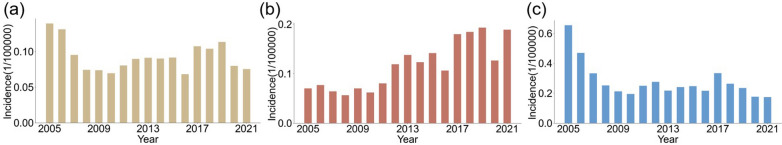
Temporal dynamics of city-level HFRS incidence rate (median values) in China (2005–2021). **a** All cities; **b** increasing-trend cities (AAPC > 0); **c** Decreasing-trend cities (AAPC < 0). HFRS incidence: incidence of hemorrhagic fever with renal syndrome; AAPC: the average annual percent change.

Regarding long-term spatiotemporal trends, China’s HFRS epidemics displayed distinct regional Heterogeneity at the city level. Between 2005 and 2021, HFRS cases occurred in 314 cities across China, with clear spatial clustering observed in Northeast, North, East, and Central China (Fig. S1). Quantitative trend analysis (Fig. S2, Fig. 1 and Table 2) classified these cities into three statistically distinct categories: stable (AAPC = 0, 4%), escalating (AAPC > 0, 40%), and declining (AAPC < 0, 56%) incidence groups, collectively indicating obvious nationwide mitigations of HFRS transmission. Meanwhile, a substantial majority of cities exhibited declining HFRS incidence in three regions: 94% of Northeast China (34/36 cities), 78% of North China (28/36 cities), and 58% of East China (45/77 cities) demonstrated obvious declining trends. Conversely, emerging upward trajectories (AAPC > 0) predominated in Northwest China (61%, 25/41 cities) and South China (57%, 20/35 cities), indicating persistent HFRS transmission hotspots in these regions. By contrast, Central China (23 cities, 52%) and Southwest China (23 cities, 54%) demonstrated a near parity between cities with downward and upward trends, with decline-to-increase ratios of 1.09∶1 and 1.15∶1 respectively (Table 2). Results demonstrated a clear north–south divergence: upward HFRS trajectories predominated in southern China—specifically the South and Southwest regions—whereas persistent downward trends characterized northern China, encompassing the Northeast, North, and Northwest zones.

**Table 2:** Spatial stratification of Chinese cities by HFRS incidence trends: Upward (AAPC > 0) versus Stable (AAPC = 0) versus Downward (AAPC < 0)

Divisions	AAPC > 0	AAPC = 0	AAPC < 0	Sum
North China	6	2	28	36
Northeast China	2	0	34	36
Northwest China	25	7	9	41
East China	32	0	45	77
Central China	21	0	23	44
Southwest China	20	2	23	45
South China	20	1	14	35
Sum	126	12	176	314

HFRS incidence: incidence of hemorrhagic fever with renal syndrome; AAPC: the average annual percent change

### Association between HFRS incidence and selected factors

**Fig.2:**
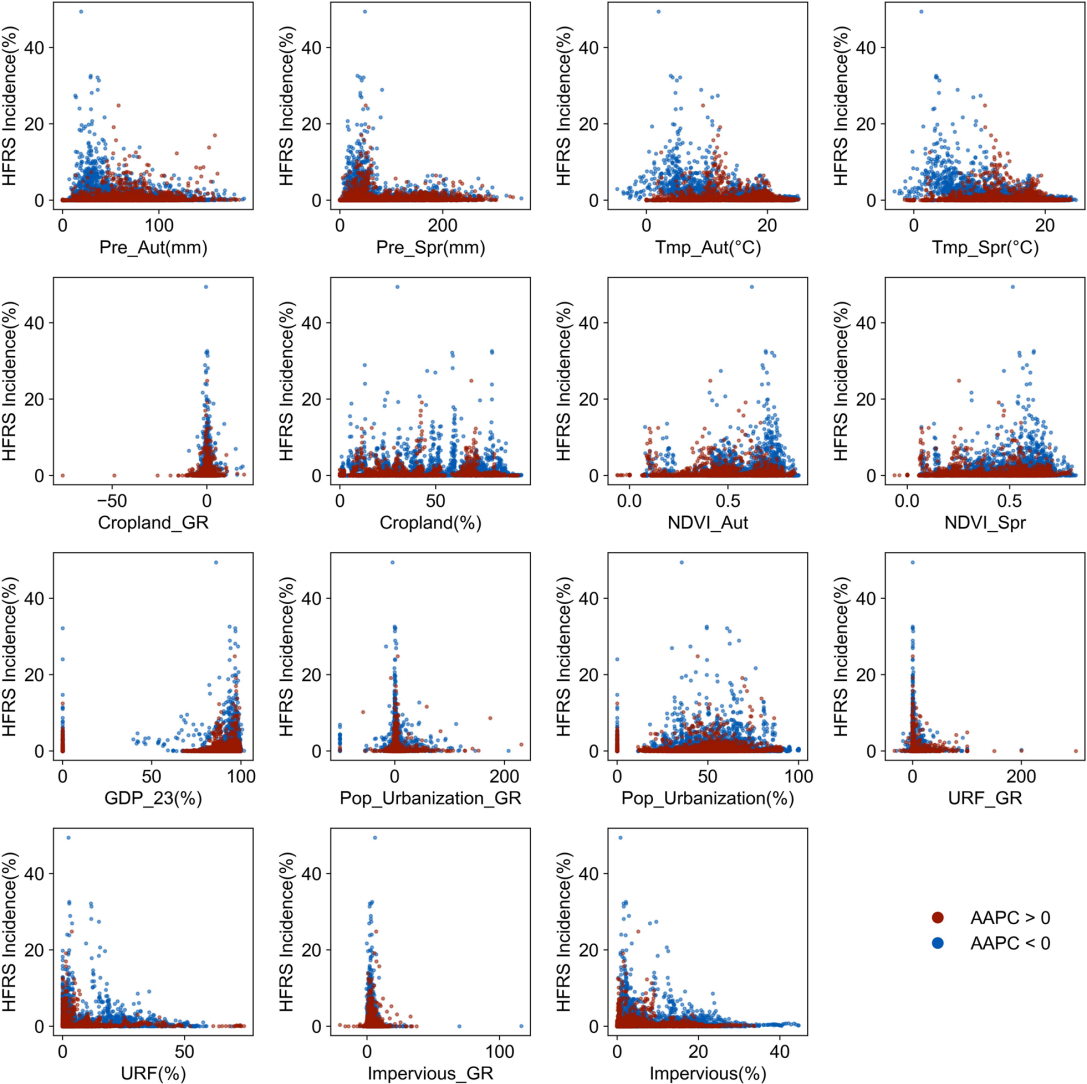
Scatter plot of HFRS incidence versus potential influencing factors in AAPC-stratified city subgroups: Upward (AAPC > 0) versus Downward (AAPC < 0). HFRS incidence: incidence of hemorrhagic fever with renal syndrome; AAPC: the average annual percent change; Prt_Aut: mean autumn precipitation; Prt_Spr: mean spring precipitation; Tmp_Aut: mean autumn temperature; Tmp_Spr: mean spring temperature; GDP_23: GDP share of secondary and tertiary sectors; Pop_Urbanization_GR: growth rate of resident population urbanization rate; Pop_Urbanization: urbanization rate of the resident population; URF_GR: growth rate of urban-rural fringe area; URF: percentage of urban–rural fringe area; Impervious_GR: growth rate of impervious area; Impervious: percentage of impervious area; Cropland_GR: growth rate of cropland area; Cropland: percentage of cropland area; NDVI: normalized difference vegetation index; NDVI_Aut: mean autumn NDVI; NDVI_Spr: mean spring NDVI

**Fig.3:**
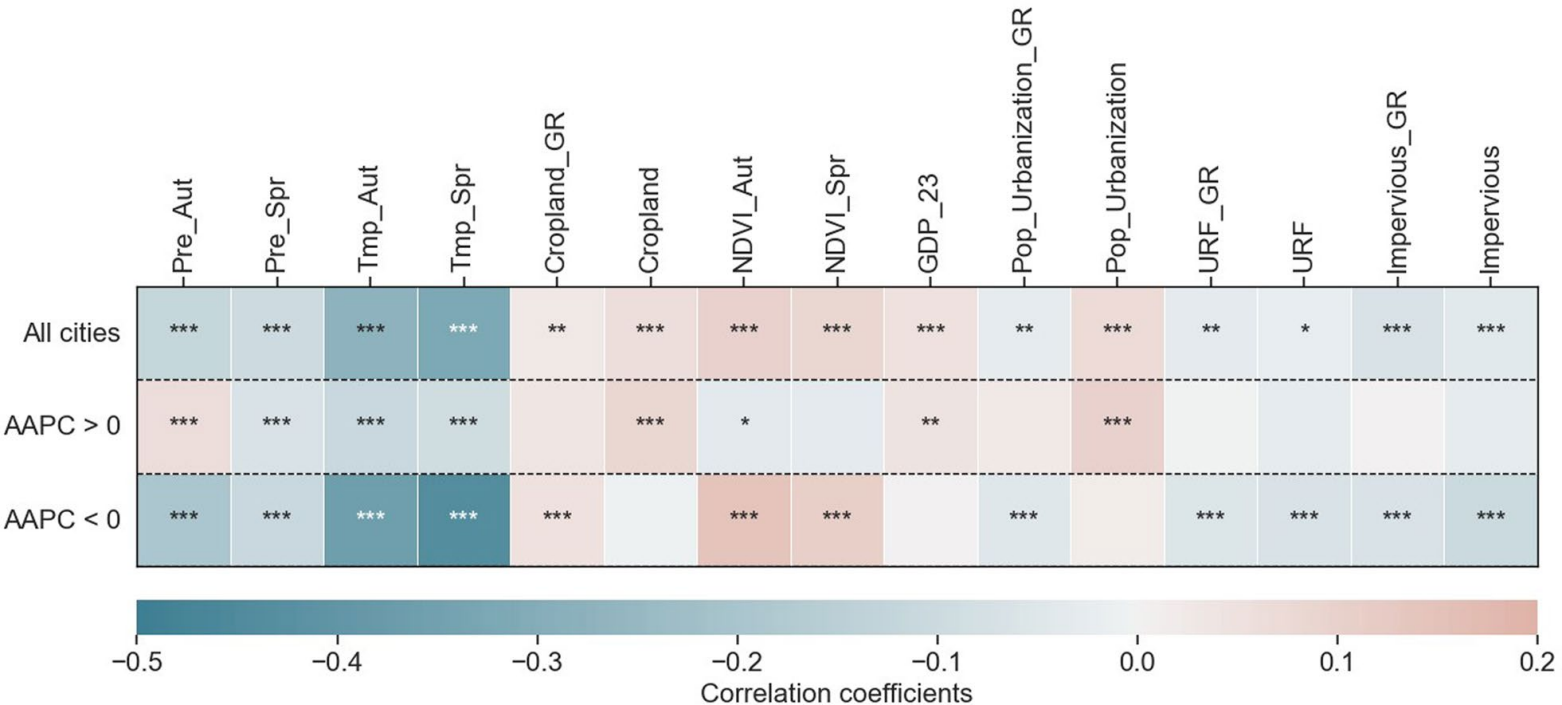
Dependent-independent variable correlations: Total cities versus AAPC-stratified city subgroups (AAPC > 0; AAPC < 0). ***, **, and * indicate the significance at the 0.01, 0.05, and 0.1 levels, respectively. No markers denote non-significant. HFRS incidence: incidence of hemorrhagic fever with renal syndrome; AAPC: the average annual percent change; Prt_Aut: mean autumn precipitation; Prt_Spr: mean spring precipitation; Tmp_Aut: mean autumn temperature; Tmp_Spr: mean spring temperature; GDP_23: GDP share of secondary and tertiary sectors; Pop_Urbanization_GR: growth rate of resident population urbanization rate; Pop_Urbanization: urbanization rate of the resident population; URF_GR: growth rate of urban–rural fringe area; URF: percentage of urban-rural fringe area; Impervious_GR: growth rate of impervious area; Impervious: percentage of impervious area; Cropland_GR: growth rate of cropland area; Cropland: percentage of cropland area; NDVI: normalized difference vegetation index; NDVI_Aut: mean autumn NDVI; NDVI_Spr: mean spring NDVI

During 2005–2021, correlation coefficients linking HFRS incidence with potential influencing factors exhibited significant variations between (1) all cities, (2) cities with upward trends (AAPC > 0), and (3) cities with downward trends (AAPC < 0) (Fig. 2 and Fig. 3). Most climatic variables presented significant negative associations (*P* < 0.01) with the city-level HFRS incidence (Fig. 3), with mean autumn precipitation (Prt_Aut) demonstrating a significant positive impacts (r = 0.07, *P* < 0.01) in upward-trend cities. As illustrated in Fig. 2, Prt_Aut concentrated within 50–100 mm in upward-trend cities, whereas downward-trend cities predominantly documented 0–50 mm (*r* = − 0.19, *P* < 0.01). Meanwhile, cropland/vegetation-related factors including the percentage of cropland area (Cropland), growth rate of cropland area (Cropland_GR), mean autumn NDVI (NDVI_Aut), and mean spring NDVI (NDVI_Spr) exhibited divergent correlations with HFRS incidence in all cities versus the two AAPC-stratified clusters (Fig. 3). Across all cities, these four variables consistently demonstrated significant positive impacts on HFRS incidence (*P* < 0.05). In upward-trend cities, cropland proportion was predominantly < 75% (*r* = 0.08, *P* < 0.01) and NDVI_Aut was significantly lower (*r* = − 0.04, *P* < 0.1). In contrast, in downward-trend cities, cropland proportion typically exceeded 50% (*r* = − 0.01, *P* > 0.1) while NDVI_Aut was markedly higher and demonstrated a significant positive correlation (*r* = 0.15, *P* < 0.01).

Similarly, urbanization-related factors also exerted divergent impacts on HFRS incidence at the city scale. Among all cities, HFRS incidence correlated positively with urbanization rate of the resident population (Pop_Urbanization, *r* = 0.10, *P* < 0.01), but inversely with five urbanization metrics (*P* < 0.01). In upward-trend cities, only GDP share of secondary and tertiary sectors (GDP_23) and Pop_Urbanization exhibited positive correlations. In downward-trend cities, all urbanization variables demonstrated strongly negative correlations (*P* < 0.01), with the exception of GDP_23 and Pop_Urbanization which showed neutral effects (*P* > 0.1). These results showed that environmental factors maintained stable spatiotemporal associations with HFRS incidence across divergent city groups, whereas urbanization drivers exhibited context-dependent heterogeneity in their relationships.

### Individual effects of potential influencing factors

**Fig.4:**
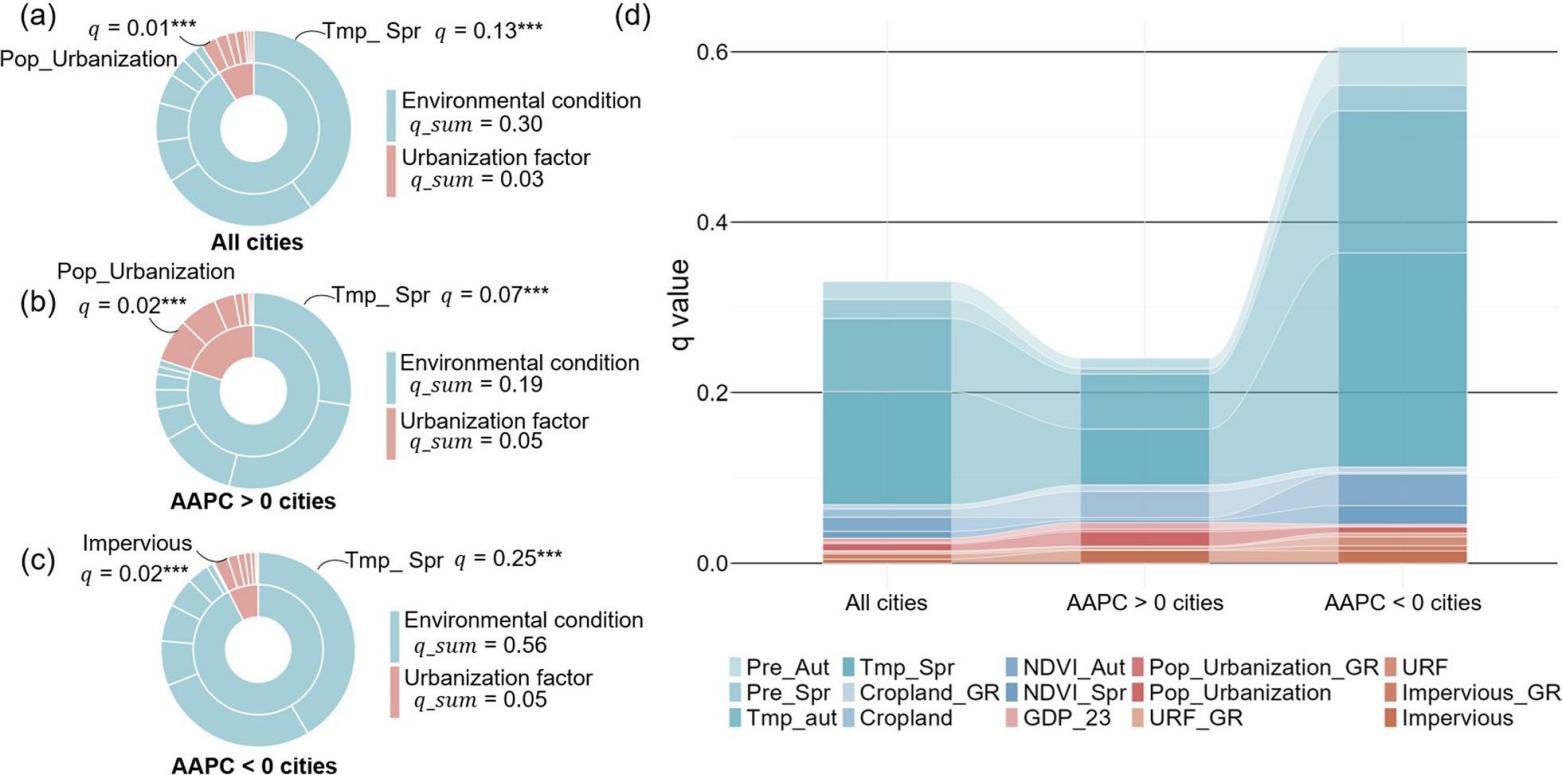
Individual explanatory power (*q* value) of independent variables. **a** All cities; **b** Upward-trend cities (AAPC > 0); **c** Downward-trend cities (AAPC < 0); **d** Variable contributions to individual explanatory power. *** indicates the significance at the 0.01 level. *q*_sum means the sum of the *q* values of all variables in this category. HFRS incidence: incidence of hemorrhagic fever with renal syndrome; AAPC: the average annual percent change; Prt_Aut: mean autumn precipitation; Prt_Spr: mean spring precipitation; Tmp_Aut: mean autumn temperature; Tmp_Spr: mean spring temperature; GDP_23: GDP share of secondary and tertiary sectors; Pop_Urbanization_GR: growth rate of resident population urbanization rate; Pop_Urbanization: urbanization rate of the resident population; URF_GR: growth rate of urban-rural fringe area; URF: percentage of urban-rural fringe area; Impervious_GR: growth rate of impervious area; Impervious: percentage of impervious area; Cropland_GR: growth rate of cropland area; Cropland: percentage of cropland area; NDVI: normalized difference vegetation index; NDVI_Aut: mean autumn NDVI; NDVI_Spr: mean spring NDVI

Geodetector analysis revealed context-dependent heterogeneity in the explanatory power (*q*-statistic) of influencing factors on city-level HFRS incidence across divergent city groups. As quantified in Fig. 4a–c, environmental factors consistently exceeded urbanization variables in individual explanatory power for HFRS incidence, with much higher *q*_sum values (environmental: 0.19 < *q*_sum < 0.56; urbanization: 0.03 < *q*_sum < 0.05). However, urbanization factors accounted for a significantly greater proportion (25%) of explanatory power for city-level HFRS incidence in upward-trend cities relative to all-city (9%) and downward-trend cohorts (8%). Among all influencing factors, mean spring temperature (Tmp_Spr) consistently exerted the strongest explanatory power for HFRS incidence across all city groups: all-city (*q* = 0.13), upward-trend (*q* = 0.07), or downward-trend (*q* = 0.25) cities at the significance level of 0.01.

As far as urbanization drivers were concerned, Pop_Urbanization exhibited moderately higher explanatory power in all-city (*q* = 0.01) and upward-trend cities (*q* = 0.02). Conversely, land urbanization rate indexed by the percentage of impervious area (Impervious) emerged as the dominant urbanization predictor (*q* = 0.02) in downward-trend cities. These analysis indicated that spatial variations in city-level HFRS incidence tended to be predominantly affected by environmental factors across China.

### Pairwise interactions of potential influencing factors

**Fig. 5:**
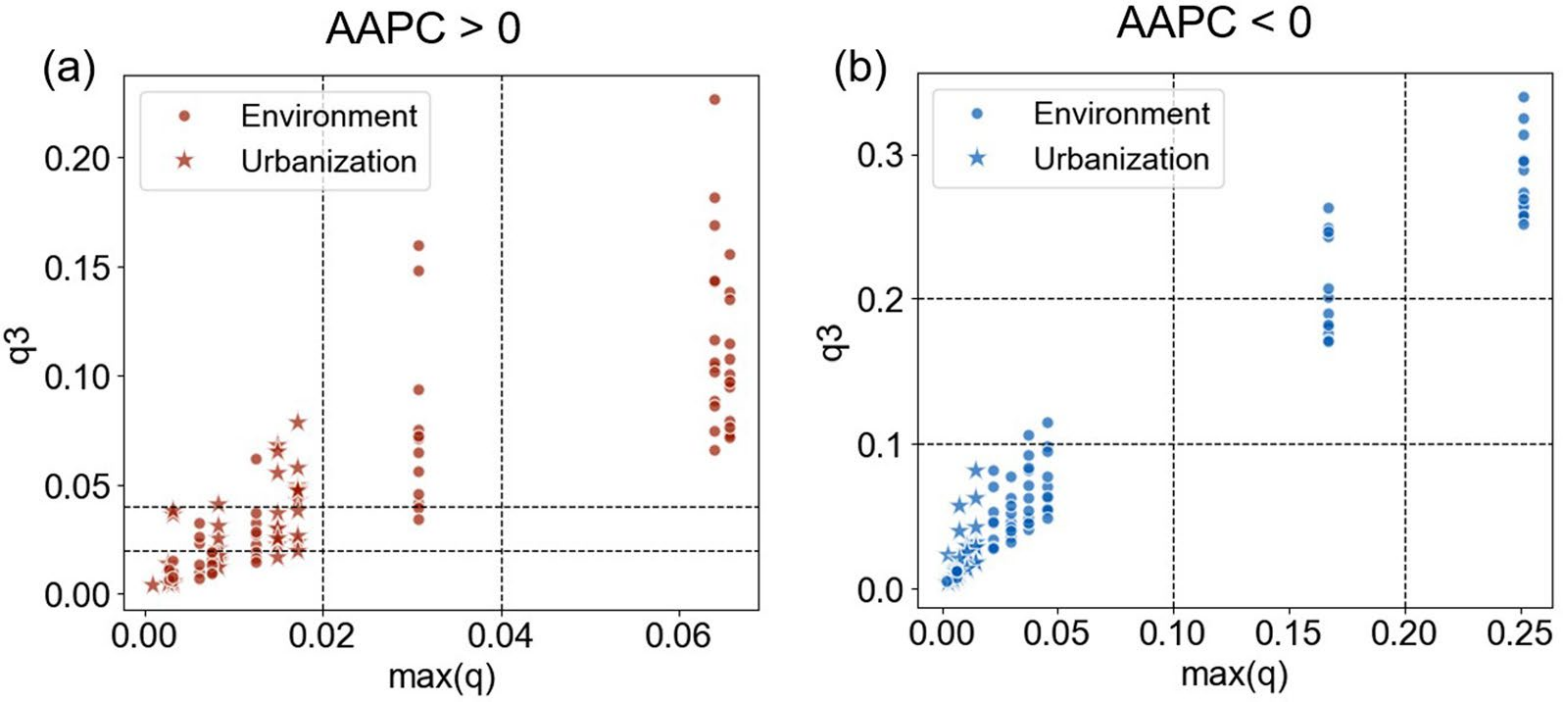
Enhanced explanatory power (*q*3) of interactions and Max (*q*1, *q*2) of independent variables in **a** upward-trend cities (AAPC < 0); **b** downward-trend cities (AAPC < 0). AAPC: the average annual percent change

As illustrated in Fig. 5, synergistic interaction between paired drivers amplified their explanatory power for city-level HFRS incidence, as evidenced in scatterplot of their maximal *q* and interaction effects (*q*3). Interaction effects diverged obviously between upward- and downward-trend cities. In upward-trend cities, 66% of factor combinations exhibited pre-interaction explanatory power (Max *q*) below 0.02, among which 35 combinations (53%) showed synergistic amplification (*sq*) ranging from 17% to 1,134%. In downward-trend cities, 74% of variable combinations displayed lower pre-interaction explanatory power (Max *q*) than 0.10, and only 46% (6/13) of combinations with Max *q* values of 0.10–0.20 demonstrated distinct interaction-driven enhancement. Comparative analysis revealed that the enhancement of interactions’ explanatory power tended to occurred in upward-trend cities.

Among the eight optimal combinations identified in Table 3, temperature factors including mean autumn temperature (Tmp_Aut) and Tmp_Spr emerged as pivotal synergistic catalyst in upward- and downward-trend cities, collectively amplifying explanatory power for city-level HFRS incidence. Meanwhile, urbanization-related interactions (Tmp_Aut paired with Imperious, GDP_23, and Pop_Urbanization) in upward-trend cities demonstrated substantial synergistic amplification (124%–184%), compared to the interactions between Tmp_Spr and percentage of urban-rural fringe area (URF), GDP_23, and Pop_Urbanization in downward-trend cities (7%–18%). These results indicated that synergistic interactions between temperature and urbanization factors have significantly amplified their explanatory power for HFRS incidence at the municipal scale.

**Table 3:** The optimal combinations with the highest explanatory power of interactions

City groups		Environment	Urbanization dimensions		
			Land use	Economy	Population
AAPC > 0	Combinations	Tmp_Aut and Cropland	Tmp_Aut and Impervious	Tmp_Aut and GDP_23	Tmp_Aut and Pop_Urbanization
	Max (*q*)	0.06	0.006	0.06	0.06
	*q*3	0.23	0.18	0.14	0.17
	*sq*	254%	184%	124%	164%
AAPC < 0	Combinations	Tmp_Spr and NDVI_Spr	Tmp_Spr and URF	Tmp_Spr and GDP_23	Tmp_Spr and Pop_Urbanization
	Max (*q*)	0.25	0.25	0.25	0.25
	*q*3	0.34	0.29	0.27	0.30
	*sq*	35%	15%	7%	18%

AAPC: the average annual percent change; Tmp_Aut: mean autumn temperature; Tmp_Spr: mean spring temperature; Cropland: percentage of cropland area; Impervious: percentage of impervious area; GDP_23: GDP share of secondary and tertiary sectors; Pop_Urbanization: urbanization rate of the resident population; NDVI_Spr: mean spring NDVI; URF: percentage of urban–rural fringe area

## Discussion

Given the persistent complexity of China’s HFRS epidemiological landscape over recent decades, characterizing comprehensive patterns of city-level HFRS incidence is essential to identify crucial influencing factors. Our study analyzed nationwide spatiotemporal dynamics of HFRS incidence at the municipal scale and identified key drivers using Joinpoint regression and Geodetector models. Several notable findings were achieved and could offer actionable insight for developing evidence-based interventions against HFRS transmission.

Many epidemiological investigation have demonstrated that the overall trend of HFRS prevalence in Chinese mainland has been declining over the past few decades [3, 38]. Our study achieved similar findings, while further revealing persistent epidemiological complexity in China’s HFRS landscape with distinct clusters of upward- and downward-trend cities shaping regional heterogeneities. This divergence may be primarily attributed to two factors: (1) nationwide implementation of preventive vaccination programs, particularly in historical HFRS hotspots such as Northeast and North China [4, 27]; and (2) heterogeneous climatic/geographic conditions coupled with regional socioeconomic disparities across the country [14, 39]. Hence, it can be seen that spatiotemporal variations of city-level HFRS incidence across China were obviously featured by an overall decline and regional differentiation. Accordingly, we recommend that at the national level, when guiding various regions to formulate HFRS prevention and control strategies, it is essential to fully recognize and uphold an overall decline and regional differentiation.

China’s HFRS transmission exhibited pronounced spatiotemporal heterogeneity, with earlier research predominantly focusing on environmental determinants including climatic variables and vegetation dynamics, which critically drive transmission variation through host-specific ecological modulation and differential human exposure pathways [2, 9, 14, 16]. Similarly, our analysis validated that both univariate associations (e.g., spring/autumn temperature, cropland proportion, and vegetation indices) and pairwise interaction effects exhibited significant explanatory power for city-level HFRS spatiotemporal heterogeneity across epidemic trajectories. Crucially, this study advanced understanding of urbanization drivers (e.g., Impervious surface rate, urbanized population proportion), demonstrating that their synergistic interactions with environmental variables significantly enhanced explanatory power for city-level HFRS incidence, especially in upward-trend cities (AAPC > 0). These findings might help account for the recent surge in HFRS incidence in rapidly urbanizing zones (e.g., Shaanxi and Hunan), where urban development fragmented natural habitats and reshaped social structures, thereby altering the ecological niches and human contact dynamics of rodent reservoir host [40–42]. It should be noted that urbanization is the driving force behind socio-economic development in different regions in the past, present, and future. Therefore, we suggest that urbanization drivers should be heavily integrated into China’s HFRS surveillance systems and targeted intervention strategies.

As an inherently multifaceted process encompassing economic restructuring, land-use intensification, and demographic reconfiguration, urbanization exhibited significant phase-dependent heterogeneity and pronounced spatial divergence across China, reflecting distinct regional development trajectories and socioeconomic gradients. In previous studies focusing on urbanization-HFRS linkages [2, 3, 14, 16, 29], much more attentions were commonly paid to the land-use dimension due to its direct modulation effects on rodent reservoir habitats and human exposure pathways. In comparison, our study extended beyond land-use intensification (impervious surface rate, URF) to incorporate socioeconomic drivers (GDP_23, Pop_Urbanization), demonstrating that urbanization factors had non-negligible impacts on HFRS spatiotemporal heterogeneity, especially in upward-trend cities (AAPC > 0). Thus, we recommend that cities, when implementing targeted interventions on this disease, should not only take into account local epidemiological characteristics (especially in cities where the disease remains at a high level or is on the rise), but also consider the multi-dimensional features of urbanization and their phased and zonal differences.

Several limitations are worth noting. First, assessment of urbanization drivers, in particular city-level public health infrastructure, was restricted by data scarcity, necessitating future integration of structured proxies (e.g., hospital bed density per capita, vaccination coverage rates) to enhance model robustness. Second, the scarcity of granular epidemiological data constrained current mechanistic analysis of urbanization-HFRS dynamics, which would be well addressed by obtaining and integrating zoonotic reservoir surveillance metric (e.g., rodent density, seroprevalence rates) in the future. Finally, high-resolution spatiotemporal data (e.g., monthly or weekly epidemiological metrics and village/township-scale influencing factors) are critical for designing precision interventions within urban area in the future although current findings are appropriate for formulating macro-level prevention and control strategies.

## Conclusions

In summary, urbanization exerts non-negligible impacts on China’s HFRS epidemic although regional disparities of this disease remain predominantly driven by climatic and ecological determinants. This research has improved our understanding of the situation of HFRS epidemics and their influencing factors in China, providing valuable insight for health authorities seeking to enhance their intervention capabilities against this disease.

## Data Availability

The datasets used and analyzed during the current study are available from the corresponding author on reasonable request.

## Abbreviations

HFRS: Hemorrhagic fever with renal syndrome
APC: The annual percent change
AAPC: The average annual percent change
Prt_Aut: Mean autumn precipitation
Prt_Spr: Mean spring precipitation
Tmp_Aut: Mean autumn temperature
Tmp_Spr: Mean spring temperature
GDP_23: GDP share of secondary and tertiary sectors
Pop_Urbanization_GR: Growth rate of resident population urbanization rate
Pop_Urbanization: Urbanization rate of the resident population
URF: Percentage of urban-rural fringe area
URF_GR: Growth rate of URF area
Cropland: Percentage of cropland area
Cropland_GR: Growth rate of cropland area
NDVI: Normalized difference vegetation index
NDVI_Aut: Mean autumn NDVI
NDVI_Spr: Mean spring NDVI
